# Wholesome Foods

**Published:** 1876-01

**Authors:** 


### WHOLESOME FOODS.

The Health Food Company still continues in its useful work of supplying unexceptionable foods to those who are sick and desire to be made well, and those who are strong and prefer to remain so. They are our neighbors at 137 Eighth Street, and we watch them closely. If they watch us as narrowly, they will notice that we make a delicious lunch daily on GRANULATED WHEAT BISCUIT AND POMARIUS. We find great comfort in these foods, and we are sure they will not deprive us of that comfort. They are pleasant to the taste, and splendid for the stomach and digestion. We wish every sick and worn-out person in the land could have the pleasure of testing these foods.

Then there is the Cold-Air Attrition Flour,—a perfect flour of the choicest wheat, uninjured by grinding between gritty millstones revolving at a rate to induce destructive heat; and not at all devitalized by any bolting process. It is a *fine* flour, though not a pure white flour. From it delicious bread and cake are made by simply *stirring* the mass,—kneading does it harm. A pound of this Cold-Air Flour is worth a half-dozen pounds of the best white flour in the market.

A splendid food, too, is the PEARLED WHEAT,—which is merely the best White Winter Wheat, with the thin, woody, outer hull, removed. It is greatly preferable to the best cracked or crushed wheats, and is also much more agreeable to the palate, being free from rough and irritating particles. It is a fact that many delicate stomachs can not bear the harsh food called "Graham," "Grits," &c. Such suffer no discomfort from the use of the cold-air flour and the Pearled Wheat,—the choice products, of Mr. Warren's Cold-Air Attrition Mills.

In answer to inquiries we would say that these products are sold in this vicinity only by the Health Food Company in New York, and at 100 Fulton Street, Brooklyn.

---

### DR. COOPER'S PADS AND BELTS.

There is a mystery about the action of these devices. That they do good seems apparent; for quite a number of our friends assert, in very positive language, that great benefit has resulted from the use of the pads and belts. It is fashionable now-a-days to wear chest-protectors of flannel and chamois, in cold weather. These appliances of Dr. Cooper are thus used, and have the double advantage of being supplied with some simple and effectual medicinal agents, which are quilted in between the soft leather and the red flannel. Through the absorption of the finely comminuted remedies thus applied, diseased conditions are neutralized and overcome. Nervous, headachy, and neuralgic people, seem to experience relief from them, and catarrhal and bronchial sufferers speak warmly of benefits received. These pads and belts are on sale at all the drug-stores of the Messrs. Hegeman.

